# Mouse-to-Human Transmission of Variant Lymphocytic Choriomeningitis Virus

**DOI:** 10.3201/eid1303.061141

**Published:** 2007-03

**Authors:** Sébastien Emonet, Karine Retornaz, Jean-Paul Gonzalez, Xavier de Lamballerie, Rémi N. Charrel

**Affiliations:** * Université de la Méditerranée, Marseille, France; † Assistance Publique–Hôpitaux de Marseille Nord, Marseille, France; ‡ Institut de Recherche pour le Développement, Bangkok, Thailand; § Assistance Publique–Hôpitaux de Marseille Timone, Marseille, France

**Keywords:** arenavirus, zoonosis, human, meningitis, encephalitis, infection, Arenaviridae, emerging

## Abstract

A case of lymphocytic choriomeningitis virus (LCMV) infection led to investigation of the reservoir. LCMV was detected in mice trapped at the patient's home, and 12 isolates were recovered. Genetic analysis showed that human and mouse LCMVs were identical and that this LCMV strain was highly divergent from previously characterized LCMV.

Lymphocytic choriomeningitis virus (LCMV) belongs to the genus *Arenavirus* in the family *Arenaviridae*. *Mus musculus* mice constitute the reservoir of LCMV in nature (*1*), but hamsters can also carry the virus. Humans usually become infected through direct contact with infected rodents or by inhaling infectious rodent excreta or secreta during occupational exposure (laboratory workers, rodent sellers) or when caring for rodents as pets. Although LCMV infection is usually asymptomatic or mild and self-limiting, it can be severe and manifest itself as meningitis and encephalitis (*2*,*3*). Infection during pregnancy may cause abortion or congenital malformations (*4*). In 2003 and 2005, two episodes of infections occurred in 2 groups of 4 recipients of solid-organ transplants; 7 of the 8 patients died (*5*). Although laboratory evidence of LCMV infection was obtained from the 8 organ recipients and from the hamster handled by donor 2, no laboratory evidence of infection could be obtained from specimens from both donors. Therefore, evidence for transmission from donor to recipient was mainly based on epidemiologic data and on the genetic identity of the virus detected in transplanted organs.

## The Study

A 5-year-old boy was admitted to the neuropediatric ward of a Marseille University hospital in August 2004 for aseptic meningitis. Forty-five days later, he was admitted again with fever and meningitis, and his condition rapidly deteriorated with encephalitis and hydrocephalus developing as described (*6*). LCMV infection was suspected on the basis of the clinical signs and a detailed interview of the mother, in which she indicated that “the house was invaded with mice.” LCMV was diagnosed based on seroconversion and positive PCR results with confirmatory sequence data (*6*).

Because *M. musculus* is the natural host of LCMV, the Public Health Office of Marseille organized a trapping campaign in the vicinity of the patient’s house, which resulted in the capture of 20 mice (*M. musculus*) with glue traps. Most were alive when they were received at the laboratory. They were humanely killed and dissected; kidneys, lungs, heart, spleen, and liver were placed individually in 1.8-mL tubes and stored at −80°C. For each animal, 1 kidney was homogeneized as previously described (*7*). Recovered material was used either for virus isolation on Vero cells (detailed protocol available on request) or for total RNA isolation with the EZ1 Virus Mini Kit v2.0 on the BioRobet EZ1 Workstation (QIAGEN SA, Courtaboeuf, France) and eluted in 75 µL final volume. A total of 10 µL was tested for LCMV RNA as described herein. Three different PCR assays, targeting the nucleoprotein gene, were used. System 1 was a nested reverse transcription–PCR (RT-PCR) that used primers 1817V-LCM (5′-AIATGATGCAGTCCATGAGTGCACA) and 2477C-LCM-3′ (5′-TCAGGTGAAGGRTGGCCATACAT-3′) for the first round and primers 1902V-LCM (5′-CCAGCCATATTTGTCCCACACTTT-3′) and 2346C-LCM (5′-AGCAGCAGGYCCRCCTCAGGT-3′) for the second round. These primers were derived from those reported by Bowen et al. (*8*,*9*) and designed from the alignment of LCMV sequence data retrieved from GenBank. System 2 was a real-time RT-PCR with primers LCM_TM_NP1 (5′-TCATGTGGCARRATGTTGTG-3′) and LCM_TM_NP2 (5′-AAAAAGAAIAARGARATCACCCC-3′) together with a FRET probe LCM_MAR_NP (5′-ATGATGCAATCCATAAGTGCGCAGT-3′). System 3 was a SYBR Green real-time RT-PCR based on primers LCM_SG_NP1 (5′-TTRTCRTCYCTYYTYTCYTTYCTCAT-3′) and LCM_SG_NP2 (5′-CAGGTRACYTTYGARAAITRRAGRAA-3′). The 3 detailed RT-PCR protocols are available on request. Human cerebrospinal fluid (CSF) samples and mouse specimens were added to Vero cells. After incubation at 37°C for 7 days, cells were tested for LCMV RNA by PCR with PCR system 1.

Results of PCRs and virus isolation are presented in the Table. Criteria to consider that samples contained LCMV RNA or LCMV were 1) virus isolation or 2) positive PCR results for at least 2 systems. Genetic analyses and phylogenetic reconstruction were based on sequences flanked by primers 1902V-LCM and 2346C-LCM. These primers amplified a 445-bp PCR product (primers included) and provided a 400-nt sequence (primers excluded) used for analysis. Nucleotide alignments were performed by using ClustalX 1.81 with default parameters (*10*). Alignments included the 16 sequences determined in this study and homologous LCMV sequences retrieved from the GenBank database. Phylogenetic analysis was performed with the Jukes-Cantor algorithm for distance calculation and the neighbor-joining method for cluster reconstruction with the MEGA 2.0 program (*11*). The robustness of nodes was tested by 500 bootstrap pseudoreplications.

**Table:** Detection of LCMV in mice trapped at patient’s home and in patient CSF*

Sample	System 1		System 2	System 3	Virus isolation	Interpreted result†
	First-round PCR	Second-round PCR				
Human						
CSF 1	–	+	+	NT	NT‡	+
CSF 2	–	+	+	NT	NT	+
Mouse						
1	–	+	+	+	–	+
2	–	–	+	+	+	+
3	–	–	–	–	–	–
4	–	+	+	–	+	+
5	–	+	+	–	+	+
6	–	+	+	–	+	+
7	–	+	+	+	–	+
8	+	+	+	+	–	+
9	+	+	+	+	+	+
10	–	–	+	+	+	+
11	–	+	+	+	–	+
12§	–	+	+	+	+	+
13	–	+	+	+	+	+
14	–	–	–	–	–	–
15	–	–	+	+	+	+
16	–	+	+	+	+	+
17	–	+	+	+	–	+
18	+	+	+	+	+	+
19	–	–	+	–	+	+
20	–	+	+	+	+	+

*LCMV, lymphocytic choriomeningitis virus; CSF, cerebrospinal fluid, NT, not tested. †An animal was considered infected with LCMV if virus was isolated either in Vero cells or results of at least 2 of 3 PCRs were positive. ‡After diagnostic tests were performed from CSF, no material was available to attempt virus isolation. §Virus strain characterized by full-length genome sequencing (GenBank accession nos. DQ286931 and DQ286932 for S and L RNA, respectively).

As shown in the Table, the 2 human CSF specimens and 14 of 20 mouse samples were PCR positive. The 2 sequences obtained from human CSF specimens were 100% identical to each other. The 14 sequences representing mouse kidney specimens were almost identical (98.5% nucleotide identity) (Figure). Comparison of human and mouse sequences showed genetic identity >98% at the nucleotide level (Figure). This high level of similarity suggests that human LCMV infection was caused by transmission from the mice. All 16 sequences determined in this study either from human or rodent material had 12%–13% nucleotide heterogeneity when compared with LCMV sequences deposited in the GenBank database and with the sequence of LCMV strain manipulated in the laboratory, thus excluding the possibility of laboratory contamination. Finally, a total of 12 strains were isolated from Vero cells; 1 was selected to be characterized by full-length genome sequencing (GenBank accession nos. DQ286931 and DQ286932 for S and L RNA sequences, respectively).

**Figure:**
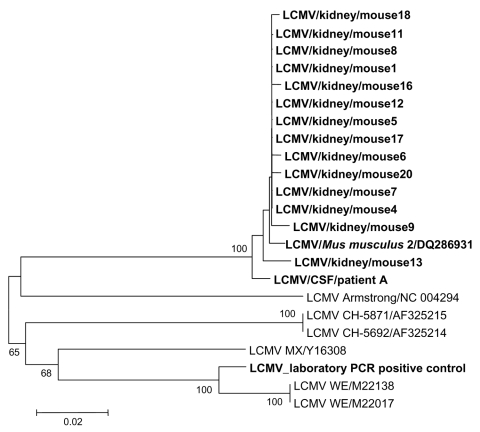
Phylogenetic tree based on 400-nt sequences amplified by PCR system 1 in the nucleoprotein gene. Lymphocytic choriomeningitis virus (LCMV) sequences characterized in this study were compared with selected homologous LCMV sequences available in the GenBank database. Sequence information corresponds to virus/nature of specimen/host/GenBank accession no. (optional), except for sequences retrieved from GenBank (virus strain/GenBank accession no.). Sequences determined in this study are in **bold type**. The 15 sequences determined from mouse material were almost identical and most closely related to LCMV sequence corresponding to the patient cerebrospinal fluid. These 16 sequences were grouped together and were clearly distinct from other LCMV strains included in the study (Armstrong, CH, MX, WE) and from Traub and Pasteur strains (data not shown).

## Conclusions

Apart from isolated cases or outbreaks of LCMV infection associated with direct contact with laboratory rodent colonies, evidence for direct epidemiologic links between human cases and wild mice was based on virus isolation and antigenic relationships. Evidence based on genetic analysis of human and mice strains was not previously reported. Field investigations, conducted between LCMV discovery in 1933 and World War II, to search for a source of human cases, reported virus isolation from gray mice (*M. musculus*) trapped in or in the close vicinity of patient’s house. The strains isolated from mice and humans were similar antigenically and immunologically. However, indisputable evidence of genetic identity was not produced. To our knowledge, this is the first report of human LCMV infection linked to wild mice as assessed by genetic evidence. Rates of infection by LCMV in wild rodent colonies reported in the literature (*12*,*13*) are of similar magnitude as the 70% rate found in this study. Such high rates can explain how clusters of human cases are likely to result from substantial exposure to infectious aerosols. In our study, the mother of the patient was negative for LCMV antibodies; other members of the family refused to undergo serologic testing. Altogether, these sequence data and the evidence of virus isolation from mice provide strong evidence that the LCMV human case resulted from infection with a virus carried by mice infesting the patient’s home through direct or indirect contact with mouse excreta. In the case reported by Fischer et al. (*5*), although the LCMV cause is not in doubt, the lack of laboratory evidence (serology, immunohistochemical staining, PCR, virus isolation) of LCMV infection in both donors is intriguing.

This study, together with recent reports of LCMV infection cases, raise concerns regarding the low level of knowledge of LCMV epidemiology that may reflect the fact that LCM was historically more prevalent in rural settings, and that it could be decreasing in the urban populations of industrialized countries. However, the growing proportion of persons living below the poverty threshold in large European and North American cities may recreate conditions compatible with the increased urban circulation of mice, and therefore increase the likelihood of rodent-associated diseases.
